# The replication origin of a repABC plasmid

**DOI:** 10.1186/1471-2180-11-158

**Published:** 2011-06-30

**Authors:** Ramón Cervantes-Rivera, Francisco Pedraza-López, Gabriela Pérez-Segura, Miguel A Cevallos

**Affiliations:** 1 Programa de Genómica Evolutiva, Centro de Ciencias Genómicas, Universidad Nacional Autónoma de México, Apartado Postal 565-A, Cuernavaca, Morelos, México

## Abstract

**Background:**

*repABC *operons are present on large, low copy-number plasmids and on some secondary chromosomes in at least 19 α-proteobacterial genera, and are responsible for the replication and segregation properties of these replicons. These operons consist, with some variations, of three genes: *repA*, *repB*, and *repC*. RepA and RepB are involved in plasmid partitioning and in the negative regulation of their own transcription, and RepC is the limiting factor for replication. An antisense RNA encoded between the *repB-repC *genes modulates *repC *expression.

**Results:**

To identify the minimal region of the *Rhizobium etli *p42d plasmid that is capable of autonomous replication, we amplified different regions of the *repABC *operon using PCR and cloned the regions into a suicide vector. The resulting vectors were then introduced into *R. etli *strains that did or did not contain p42d. The minimal replicon consisted of a *repC *open reading frame under the control of a constitutive promoter with a Shine-Dalgarno sequence that we designed. A sequence analysis of *repC *revealed the presence of a large A+T-rich region but no iterons or DnaA boxes. Silent mutations that modified the A+T content of this region eliminated the replication capability of the plasmid. The minimal replicon could not be introduced into *R. etli *strain containing p42d, but similar constructs that carried *repC *from *Sinorhizobium meliloti *pSymA or the linear chromosome of *Agrobacterium tumefaciens *replicated in the presence or absence of p42d, indicating that RepC is an incompatibility factor. A hybrid gene construct expressing a RepC protein with the first 362 amino acid residues from p42d RepC and the last 39 amino acid residues of RepC from SymA was able to replicate in the presence of p42d.

**Conclusions:**

RepC is the only element encoded in the *repABC *operon of the *R. etli *p42d plasmid that is necessary and sufficient for plasmid replication and is probably the initiator protein. The *oriV *of this plasmid resides within the *repC *gene and is located close to or inside of a large A+T region. RepC can act as an incompatibility factor, and the last 39 amino acid residues of the carboxy-terminal region of this protein are involved in promoting this phenotype.

## Background

Proteins that are involved in the initiation of DNA replication are essential to cells. These proteins recognize the origin of replication, destabilize double-stranded DNA, and recruit the replisome, which is the machinery directly involved in DNA replication [1].

Both the activity and concentration of the initiator proteins are highly regulated because the genetic material needs to be replicated only once per generation. A failure in this process could accelerate the production of new DNA molecules with a concomitant increase in the number of new origins of replication, which could be used in new rounds of replication and leading to cell death (i.e., "runaway replication") [2].

Initiator proteins control the replication rate using several mechanisms that limit either their own synthesis or their availability. The initiator proteins can directly auto-regulate the transcription of their own genes or trigger the production of negative regulators, antisense-RNAs or proteins, which are co-transcribed with the initiator genes. The activity of the initiator proteins can be controlled by covalent modifications or by titrating out their availability using DNA sites that resemble origins of replication. In addition, the DNA initiation rate can be controlled by blocking or hiding the origins of replication [3,4].

The initiation of replication of the *Escherichia coli *chromosome and of some of its plasmids has been studied extensively. However, our knowledge of other bacterial replication systems is limited. Research on new replicons that are not found in *E. coli *or its close relatives would yield new insights into the regulation of initiation of replication in bacteria. The present work concerns *repABC *replicons, which are present on large, low copy-number plasmids and on some secondary chromosomes in at least 19 α-proteobacterial genera. Some bacterial strains contain more than one *repABC *replicon, indicating that this plasmid family encompasses several incompatibility groups [5-7].

The basic replicon of *repABC *plasmids is compact because all of the elements required for replication and segregation are encoded in a single operon, the *repABC *operon [8,9]. However, this operon is controlled by a complex regulatory mechanism. The first two genes of the *repABC *operon encode for proteins belonging to a type Ia segregation system [10]. RepA and RepB have been implicated in the negative transcriptional regulation of the *repABC *operon [9,11].

RepC is a limiting replication factor and thus has been suggested to be the initiator protein [8,12,13]. The members of the *repABC *family contain a centromeric-like sequence (*parS*) in three possible locations: downstream of and close to the stop codon of *repC *[14,15], between *repA *and *repB*, or upstream of *repA *[16,17]. A conserved sequence between the *repB *and *repC *genes is present in all known *repABC *replicons and contains an antisense RNA (ctRNA) gene, the product of which negatively modulates the expression of RepC [18-20]. Regulatory role of the ctRNA depends on its pairing with the *repABC *mRNA. In the absence of the ctRNA, the mRNA section corresponding to the *repB-repC *intergenic region folds into a large stem-loop structure so that the predicted *repC *Shine-Dalgarno (SD) sequence and the *repC *initiation codon remain single-stranded, allowing *repC *translation. In contrast, when the ctRNA hybridizes with the *repABC *mRNA, the *repC *leader sequence forms an intrinsic terminator, blocking *repC *transcription [21].

Many aspects of the biology of these plasmids remain unknown, especially the details of the replication or segregation of these genetic elements. In this paper, we demonstrate the following: A) RepC is the only element encoded in the *repABC *operon of the *Rhizobium etli *p42d plasmid (formally pRetCFN42d) that is necessary and sufficient for plasmid replication. B) RepC is an incompatibility factor. C) The RepC carboxy-terminal region is involved in the incompatibility phenotype. D) The origin of replication of the *repABC *plasmid resides in a large A+T-rich region located at the central section of the *repC *gene.

## Methods

### Plasmids, bacterial strains and growth conditions

The bacterial strains and the plasmids used in this work are described in Table 1. *E. coli *strains were grown at 37°C in Luria-Bertani medium. *Rhizobium *strains were grown at 30°C in PY medium supplemented with 1 mM CaCl_2 _[22]. Nalidixic acid (20 μg/ml) and chloramphenicol (30 μg/ml) were added when required. Growth kinetics were made in 500 ml flasks containing, 50 ml of PY medium without antibiotics. Incubation was performed at 30°C and 250 rpm.

**Table 1:** Bacterial strains and plasmid used in this work

Strain	Relevant characteristics	Reference
*Rhizobium etli *CE3	Streptomycin resistant derivative of CFN42 strain	[20]
*R. etli *CFNX101	*recA*::Ω-Spectinomycin derivative of CE3	[46]
*R. etli *CFNX107	*recA*:: Ω-Spectinomycin derivative of CE3, laking plasmid p42a and p42d.	[46]
*E. coli *S17-1	Plasmid donor in conjugations	[23]
		
**Plasmid**	**Relevant characteristics**	**Reference**
pDOP	A chloramphenicol resistant suicide vector derived from pBC SK(+), and containing *oriT*	This work
pDOP-E'	pDOP derivative with the intergenic region *repB-repC*, the complete *repC *gene under Placpromoter, and 500 pb downstream repC stop codon.	[22]
pDOP-H3	pDOP derivative carrying a 5.6 Kb *Hin*dIII with *repABC *operon of *R. etli *plasmid p42d.	This work
pDOP-αC	pDOP derivative with the intergenic region *repB-repC *and the complete repC gene under Plac promoter.	This work
pDOP-C	pDOP carrying repC gen of plasmid p42d, with a SD sequence (AGGA) and under Plac promoter.	This work
pDOP-C/D1UM	Similar to pDOP-C but with a *repC *gene carrying a deletion from codon 2 to codon 29	This work
pDOP-C/RD1L	Similar to pDOP-C but with a *repC *gene carrying a deletion from codon 372 to codon 401	This work
pDOP-F1	pDOP containing a *repC *fragment from codon 2 to codon 110, with a SD consensus sequence under Plac promoter.	This work
pDOP-C/F1-F2	pDOP containing a *repC *fragment from codon 2 to codon 209, with a SD consensus sequence under Plac promoter.	This work
pDOP-C/F1-F3	pDOP containing a *repC *fragment from codon 2 to codon 309, with a SD consensus sequence under Plac promoter.	This work
pDOP-C/F4	pDOP containing a *repC *fragment from codon 310 to codon 403, with a SD consensus sequence under Plac promoter.	This work
pDOP-C/F4-F3	pDOP containing a *repC *fragment from codon 210 to codon 403, with a SD consensus sequence under Plac promoter.	This work
pDOP-C/F4-F2	pDOP containing a *repC *fragment from codon 111 to codon 403, with a SD consensus sequence under Plac promoter.	This work
pDOP-C s/SD	Similar to pDOP-C but without the SD sequence	This work
pDOP-TtMC	Similar to pDOP-C but with a mutant *repC *gene carrying	This work
	silent mutations to increase its CG content	
pDOP-CBbglll	Similar to pDOP-C but with *repC *gene, carrying a frameshift mutation at the *Bgl*II restriction site	This work
pDOP-CSphI	Similar to pDOP-C but with *repC *gene, carrying a frameshift mutation at the *Sph*I restriction site	This work
pDOP-CAtLC	pDOP derivative carrying *repC *gen of the *Agrobacterium*	This work
	*tumefaciens *C58 linear chromosome, with a SD sequence (AGGA) and under Plac promoter.	
pDOP-CsA	pDOP derivative carrying *repC *gen of the *Sinorhizobium meliloti *1021 pSymA, with a SD sequence (AGGA) and under Plac promoter.	This work
pDOP/C420-1209	pDOP with a hybrid *repC *gene, encoding the first 140 amino acid residues of the pSymA RepC protein and the rest of p42d.	This work
pDOP/C1-420	pDOP with a hybrid *repC *gene, encoding the first 140 amino acid residues of the p42d RepC protein and the rest of pSymA.	This work
pDOP/C421-840	pDOP with a hybrid *repC *gene encoding, the first 140 amino acid residues of the pSymA RepC protein, the next 140 amino acid residues from the p42d RepC protein and the rest from the pSymA RepC protein.	This work
pDOP/Cs421-840	pDOP witha hybrid *repC *gene, encoding the first 140 amino acid residues of the p42d RepC protein, the next 140 amino acid residues from the pSymA RepC protein and the rest from the p42d RepC protein.	This work
pDOP/C841-1209	pDOP derivative with a hybrid *repC *gene, encoding the first 280 aminoacid residued of pSymA RepC and the rest of p42d RepC protein.	This work
pDOP/C1-990	pDOP derivative with a hybrid *repC *gene, encoding the first 330 amino acid residues of p42d RepC protein and the rest of pSymA RepC protein.	This work
pDOP/C1-1086	pDOP derivative with a hybrid *repC *gene, encoding the first 362 amino acid residues of p42d RepC protein and the rest of pSymA RepC protein.	This work

### Bacterial mating

pDOP derivatives were introduced into *Rhizobium *by conjugation using *E. coli *S17-1 as a donor strain [23]. The strains were grown in the proper antibiotic-free liquid medium to stationary phase, mixed in a donor-recipient ratio of 1:2 on antibiotic-free PY plates, and incubated at 30°C overnight. The cells were resuspended in PY medium, and serial dilutions were plated on the appropriate selective PY medium.

### Constructions

The primers used in this work are presented in Table 2. The plasmid pDOP-H3 replicon was obtained by cloning a 5.6 Kb *Hin*dIII fragment containing the complete *repABC *operon from pH3 into the *Hin*dIII site of pDOP [24]. The inserts of the plasmids used in this work, unless otherwise indicated, were PCR amplified from *repABC *operon cloned in pH3 [24]. Inserts were introduced into pDOP in the sense orientation with respect to the promoter, using restriction sites that were included in the primer sequences.

**Table 2:** Oligonucleotides used in these work

Name	Sequence
ALFAU2	5'-AGGGTACCCCGCAAAAGAAAAGA
Mal-C2Kpn	5'-TCGGTACCTTACCCAGCCCTCAAACC
RBS-C	5'-GGATCCAAGGAAACAGCTATGCAGTCGGGGAATG
repC-F1U	5'-GCGGCCGCGGATCCAATGCAGTCGGGGAATGTA
repC-F1L	5'-ACTAGTCCCGGGAACCCCGACTCCACCAGA
repC-F2U	5'-GCGGCCGCGGATCCAATGGATCGTCCGTAAGGATAG
repC-F2L	5'-ACTAGTCCCGGGGCGCGGAATTCTGCTCGC
repC-F3U	5'-GCGGCCGCGGATCCAATGGTTCCGACCCTTGAAGGG
repC-F3L	5'-ACTAGTCCCGGGGCGCGGTGCATAGTCGCT
repC-F4U	5'-GCGGCCGCGGATCCAATGGGCGTGGGAAGCTGGCGA
repC-F4L	5'-ACTAGTCCCGGGTTACCCAGCCCTCAAACC
repC-D1U	5'-GGATCCAAGGAAACAGCTATGACGCTTGCGCTCGTGC
repC-D1L	5'-GCCAAGCTTTTATATCATCGGGCCAAGC
repC-D2U	5'-GGATCCAAGGAAACAGCTATGGGAAAAGCTGCCGATA
repC-D2L	5'-GCCAAGCTTTTATAGATCCCGCAGATAG
repC-D3U	5'-GGATCCAAGGAAACAGCTATGGAACTGCTCAAGA
repC-D3L	5'-GCCAAGCTTTTATTCCAGGATGCACGCA
repCd-sSDU	5'-GAAAAGAGCTCCCTCAACGT
Cd-StopC-U	5'-GGATCCTAACAGTCGGGGAATG
Ttrack1-U	5'AGCCCGAGTCCGTGAACGAGTCCGAGCCGCGCTCCGAGAAG
	GAGCAGCACATACAGAATTCAAAACCC
Ttrack1-L	5'-CTCGAGGTTCTCCAAGCGGTTCAGCACCTCCTCGCGGAG
	CATCTCCATCTCGTTGAGCACGCTAGTGACCCCTTCAAG
Ttrack2-U	5'-ATCGAGCAGCACATCCAGAACTCCAAGCCCGAGTCCGTG
Ttrack2-L	5'-GGAGTTCTGGATGTGCTGCTCGATGTGGGCGGCGTTGGT
	GCTGATCTTCTCGGCGTTCTCGAGGTTCTCCAAGCGGTT
repCATBamU	5'-GGATCCAAGGAAACAGCTATGGACAGCACATGTGTAACG
repCATHindL	5'-AAGCTTCTAACCCGCCATGCCCACCTC
K-Syma-L	5'-GGTACCTCACGACACCCCCCGCCC
C-SymA	5'-GGATCCAAGGAAACAGCTATGGAGATTGGAAGTGTGACG
Mal-C2	5'-TCAAGCTTTTACCCAGCCCTCAAACC
AL-2U	5'-ATCGGCACAGCGTTCGGCTTTTCGTCGCCCCTC CTGGCGCGATCGGAA
1L-BU	5'-GAGAATGCTTTTGGCTTCGACCTGCTCGCACCAC TGCTGGCCCGCTCC
2L-CU	5'-GAGCAGGGGGCAAAGGCGAGCTTGGAACCGGCC AACAAGGCAAAAAGG
BL-3U	5'-GAGCGGTTAGACGGCCAAGCCATTAGCCTTCAGC CGAAGAATGAATCG
AL-2Uc	5'-CGAAAAGCCGAACGCTGTGCCGAT
1L-BUc	5'-CAGGTCGAAGCCAAAAGCATTCTC
2L-CUc	5'-CAAGCTCGGCTTTGCCCCCTGCTC
BL-3Uc	5'-AATGGCTTGGCCGTCTAACCGCTC
Cd-1086L	5'-GTTGATGAAGTTAGCCCTTTCCAG
SaU-CdL	5'-AACGCCAAGCATAGATCGTACCGT CCCCAGCATAGATCGAACCACCAC
SaL-CdU	5'-CTGTTGAGCTTCTATCCGGAGAAC GAGTTGCGTCAGGATGCACAATTG
Cs-1087U	5'-CTGGAAAGGGCTAACTTCATCAA CTCTGCTGGCGGCTATCTGCGCGAT

#### Constructs used to determine the minimal replicon

Insert of plasmid pDOP-αC was generated by amplifying the *incα*-*repC *region with the primers ALFAU2 and Mal-C2Kpn. The *repC *(p42d) gene present in pDOP-C was amplified by PCR with the primers RBS-C and Mal-C2. The *repC *gene of pSymA, present in construct pDOP-CsA, was obtained by PCR with the primers C-SymA and K-Syma-L and the genomic DNA of *S. meliloti *2011 as the template. The *repC *gene of the linear chromosome of *Agrobacterium tumefaciens *C58 was obtained by PCR with the primers repCATBamU and repCATHinL and genomic DNA as the template.

#### Constructs carrying *repC* deletions or *repC* fragments

The insert of the plasmid pDOP-C/D1UM with a deletion in its 5'-end was obtained with the oligonucleotides repC-D1U and Mal-C2. The *repC *gene present in the plasmid pDOP-C/RD1L was amplified with the primers RBS-C and repC-D1L. Six plasmids carrying fragments of the *repC *gene were constructed: pDOP-C/F1 insert was obtained with primers repC-F1U and repC-F1L. The insert of plasmid pDOP-C/F1-F2 was obtained with primers repC-F1U and repC-F2L. Inserts of plasmids pDOP-C/F1-F3, pDOP-C/F4, pDOP-C/F4-F3, and pDOP-C/F4-F2 were obtained with the following primer pairs: repC-F1U and repC-F3L, repC-F4U and repC-F4L, repC-F3U and repC-F4L, repC-F2U and repC-F4L, respectively.

#### Construction of a *repC* mutant lacking its Shine-Dalgarno sequence

The insert of the plasmid pDOP-Cs/SD was acquired by PCR with the primers repCd-sSDU and Mal-C2.

#### Constructs carrying *repC* frame-shift mutations

Plasmid pDOP-CBglII, was constructed digesting pDOP-C with *Bgl*II and filling in 5'-overhangs with T4 DNA polymerase (Fermentas). The blunted plasmid was ligated again with T4 ligase (Fermentas). Plasmid pDOP-CSphI was constructed in a similar way but digesting pDOP-C with *Sph*I.

#### Construction of a *repC* gene carrying synonomous mutatios in the A+T rich region

The pDOP-TtMC insert was obtained by an overlap extension PCR as described by Horton et al. (1989) [25]. The first PCR was performed using the primers Ttrack1-U and Mal-C2Kpn, and pH3 DNA as initial template. This product was purified and used as template for a second PCR with the oligonucleotides Mal-C2Kpn and Ttrack2-U; the amplification product was named T2-U. A third PCR amplification product obtained with the primers RBS-C and Ttrack1-L, and pH3 DNA as the template, was purified and used as a template in a new PCR reaction with the primers RBS-C and Ttrack2-L. The amplification product was named T2-L.

Finally, PCR products T2-U and T2-L were then mixed and used as the template for the last PCR. In this reaction, the primers Mal-C2Kpn and RBS-C were used, and the final PCR product was cloned into pDOP.

#### Construction of *repC *hybrid genes

Overlap extension PCR was also employed to obtain *repC *hybrid genes. RepC gene amplification products from pSymA were obtained using pDOP-CsA as the template, and the *repC *p42d products were obtained using pH3 as the template. Most of the hybrid genes described here required the overlap of two PCR products. The insert of plasmid pDOP/C420-1209 was obtained using the primers C-SymA and AL-2Uc for the first PCR product and AL-2U and Mal-C2 for the second product. The final PCR product was obtained with the external primers C-SymA and Mal-C2. The insert of plasmid pDOP/C1-420 was constructed with primers RBS-C and 1L-B2c and the primers 1L-B2 and K-SymAL for the first and second PCR products, respectively. These products were combined using the primers RBS-C and K-SymAL. The pDOP/C841-1209 insert was constructed with the primers C-SymA and BL-3Uc for the first PCR product and BL-3U and Mal-C2 for the second. These products were joined in a third PCR with the primers C-SymA and Mal-C2. The hybrid gene in pDOP/C1-990 was acquired with the primers RBS-C and Sal-CdL for the first PCR product and Sal-CdU and Mal-C2 for the second. These PCR products were integrated in a third PCR with the primers RBS-C and Mal-C2. Similarly, the hybrid gene of pDOP/C1-990 was obtained with the primers RBS-C and Cd-1086 for the first amplification product. To obtain the second PCR product, the primers Cs-1087U and Mal-C2 were used, and both PCR products were fused with the primers RBS-C and Mal-C2. The inserts of two of the constructs, pDOP/C421-840 and pDOP/Cs421-840, required the fusion of three PCR products. The hybrid gene located in pDOP/C421-840 required the primers C-SymA and AL-2Uc for the first PCR product, the primers AL-2U and AL-2Uc for the second PCR product, and the primers 2L-CU and K-SymA for the third PCR product. The three PCR products were fused in the final PCR with the primers C-SymA and K-SymA. The hybrid gene present in pDOP/Cs421-840 was obtained using the primers RBS-C and 1L-B2c for the first PCR product, the primers 1L-B2 and B2-3Uc for the second PCR product, and the primers BL-2U and Mal-C2 for the third PCR product. These PCR products were linked using the primers RBS-C and Mal-C2 in the final PCR. DNA sequences of the inserts of all constructs were obtained to corroborate their correctness.

### Plasmid profiles

The plasmid profiles of four transconjugants from each cross were visualized on agarose gels according to the protocol described by Hynes and McGregor [26].

### DNA isolation and manipulation

Plasmid DNA was isolated using the High Pure Plasmid Isolation Kit (Roche) according to the manufacturer's instructions. Restriction and ligation reactions were performed under the conditions specified by the enzyme manufacturer (Fermentas). PCR was performed using Platinum High Fidelity *Taq *Platinum Polymerase or ThermalAce™ DNA Polymerase (Invitrogen). PCR products were cloned using the TOPO TA Cloning Kit (Invitrogen).

### Plasmid incompatibility

The incompatibility properties of the constructs were determined as described in Ramírez-Romero *et al*. [7].

### Plasmid replication in *R. etli*

To determine the replication capabilities of the pDOP derivatives in *R. etli*, the plasmids were introduced into CFNX107 by conjugation. The plasmid profiles of at least four transconjugants from each cross were analyzed. A recombinant plasmid was considered capable of replicating in *R. etli *if the plasmid profiles of the transconjugants showed a new band of the expected size.

### Plasmid copy-number determination

The plasmid copy numbers of the CFNX107 transconjugants containing pDOP derivatives were evaluated as follows: the total DNA of each transconjugant was isolated, digested with *Hin*dIII endonuclease, resolved in a 1% agarose gel and transferred to Hybond-N+ membranes (Amersham). The blot was then simultaneously hybridized with an Ω- spectinomycin cassette located within the *recA *gene (chromosome-encoded) and with a fragment of pDOP; both probes were of the same size and GC content. The hybridization signals were quantified with a PhosphorImager SI (Molecular Dynamics). The plasmid copy-number was calculated from the ratio of the integrated hybridization signal of the recombinant plasmid and the integrated hybridization signal of the chromosome.

### Bioinformatics

Alignments were performed with Clustal-W [27] at the WWW service of the European Bioinformatics Institute http://www2.ebi.ac.uk/clustalw. Protein secondary structure predictions were made with PSIPRED [28] at the WWW service of the Bioinformatics Group, UCL Department Of Computer Science http://bioinf.cs.ucl.ac.uk/psipred/. The DNA duplex helical stability profile was calculated using WEB-THERMODYN: sequence analysis software for profiling DNA helical stability http://www.gsa.buffalo.edu/dna/dk/WEBTHERMODYN/[29].

## Results

### The *oriV *of p42d is located within the *repC *coding sequence

**Figure 1:**
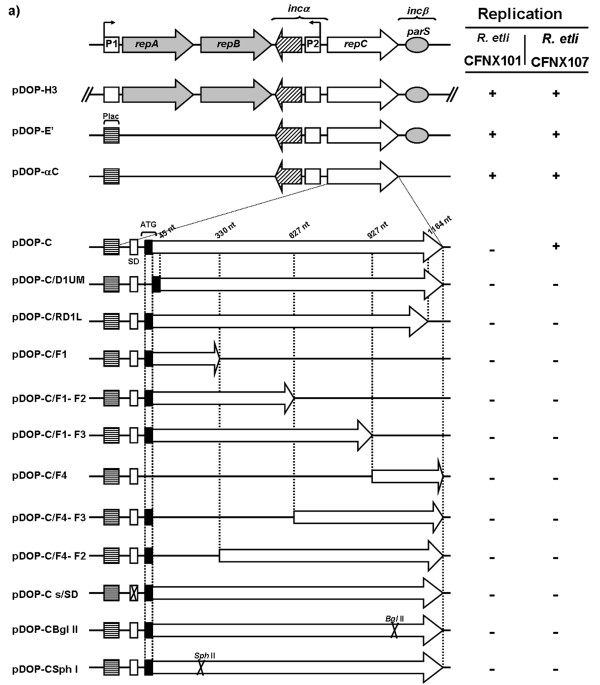
**Linear representation of the constructs used in this work**. **a) **At the top of the figure the p42d *repABC *operon is shown. Grey arrows represent genes encoding the partitioning proteins and *parS *and the grey ellipse represents the centromeric-like region *parS*. A white arrow shows the relative position of the gene encoding RepC, a protein essential for replication. Dashed arrow represents a gene encoding a small antisense RNA that modulates *repC *expression. Boxed P1 and P2, indicate the position and transcription directions of the promoters found within the *repABC *operon. Brackets indicate regions involved in plasmid incompatibility. Below, graphic representation of the genetic elements present in each one of the constructs used in this work, using the same symbols than above. Square filled with horizontal lines shows the relative position of pLac, a constitutive promoter in *Rhizobium*. **b) **A magnification of the *repC *gene and *repC *gene fragments present in the constructs, including the genetic elements introduced by us: white vertical rectangle represent a Shine-Dalgarno (SD) sequence, while the black vertical rectangle shows the initiation codon. Crossed rectangle indicates that the SD sequence was eliminated in that particular construction. Crosses within the white arrows, marked with *Sph*I or BglII, indicate that inserts of those constructs possess a frame-shift mutation in that specific point. Construct names are listed in the left column and their replication capabilities in strains CFNX101 and CFNX107 are listed in the columns in the right: (+) indicates that the construct is capable of autonomous replication and (-) that the construct does not have this property.

The basic replicon of *Rhizobium etli *p42d, defined as the smallest DNA region that contains all of the elements required to replicate with the same stability and plasmid copy-number as the parental plasmid, consists of the complete *repABC *operon plus 500 bp downstream of the *repC *stop codon (*inc*-beta region, containing *parS*) and 86 bp upstream of the *repA *initiation codon [8] (Figure 1). pDOP-E', a construct which carries the complete *repC *gene, the intergenic sequence between *repB *and *repC *(*inc-alpha*), and the 500 bp downstream of the *repC *stop codon under a constitutive promoter (Plac promoter), can replicate but does so with a slightly higher plasmid copy-number than the parental plasmid. However, derivatives that lack parts of the gene encoding the antisense RNA were unable to replicate [20].

To identify the minimal region of p42d that is capable of independent replication (putting aside the properties of the parental plasmid), we further explored the region between the *repB *stop codon and the 500 bp downstream of the *repC *stop codon. Three PCR products that possessed parts of this region were amplified and cloned into pDOP, a mobilizable suicide vector, under the control of the Plac promoter, which behaves as a constitutive promoter in *Rhizobium*. The first construct (pDOP-αC) contained the *repB-repC *intergenic region (*inc-alpha*) and the complete *repC *gene. The second construct, pDOP-SDnC, contained the *repC *open reading frame (ORF), including its putative *repC *Shine-Dalgarno (SD) sequence (AGGUG). The third construct contained the *repC *ORF but with a SD sequence that was more similar to the *Rhizobium etli *SD consensus (AGGAA) positioned 6 bp prior to the *repC *initiation codon (pDOP-C). As a control, we introduced a *Hin*dIII fragment of 5.6 Kb that carried the entire *repABC *of p42d into pDOP conferring it the ability to replicate in *Rhizobium *(Figure 1) [24].

**Figure 2:**
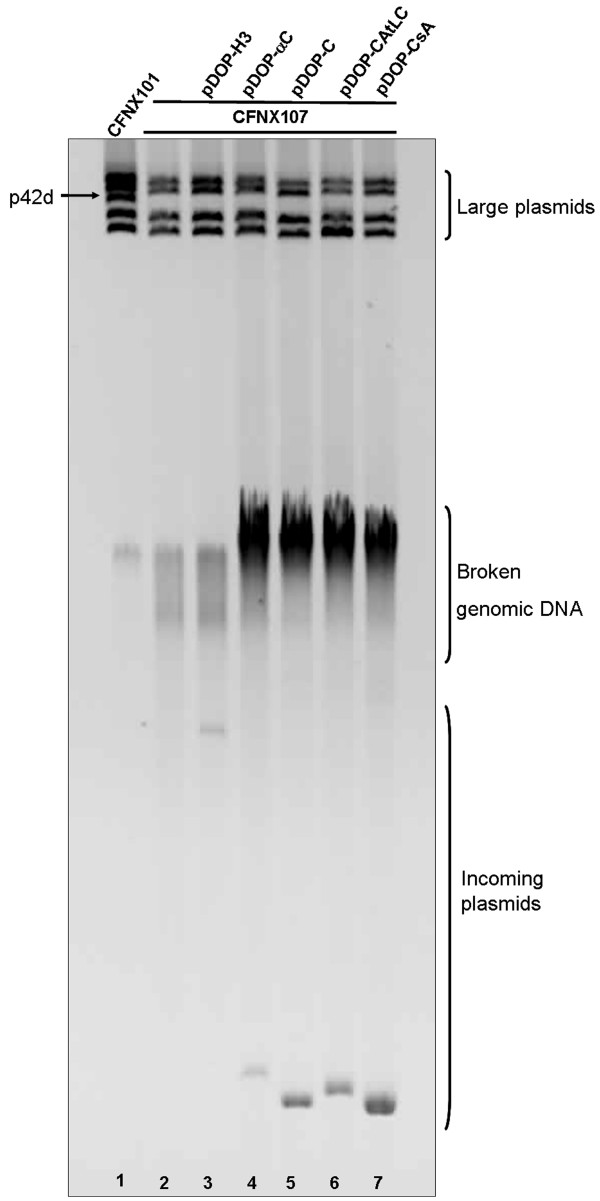
**Plasmid profiles of *Rhizobium etli *CFNX101, and *Rhizobium etli *CFNX107 transconjugants, carrying the following plasmids: pDOP-H3, pDOP-αC, pDOP-C, pDOP-CAtLC, pDOP-CsA**. Brackets at right show the positions of the resident large plasmids, broken DNA, and of the incoming plasmids. Arrow at left shows the location of plasmid p42d, in *R. etli *CFNX101. Negative image of Ethidium bromide stained gel.

**Figure 3:**
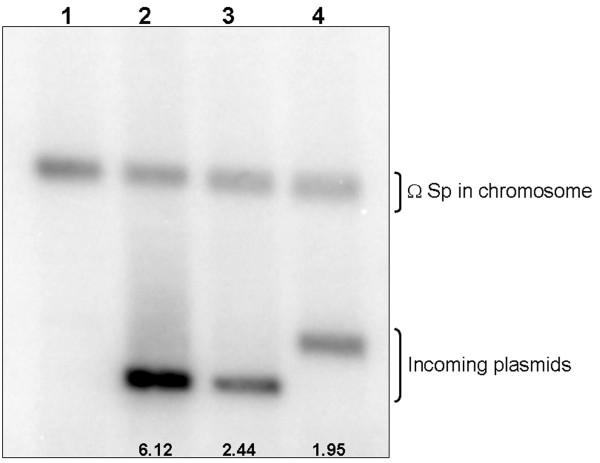
**Plasmid copy number**. Autoradiogram of a Southern blot of total DNA digested with *Hin*dIII and probed simultaneously with The Ω-Spc cassette, located within *recA *gene (chromosomal detector) and with a pDOP vector (incoming plasmid detector). The plasmid copy number of each strain was calculated as the ratio of the integrated hybridization signal of *repC *(incoming plasmid) and the integrated hybridization signal of Ω-Spc cassette (chromosome). Lane 1, CFNX107; lane 2, CFNX107/pDOP-C; lane 3, CFNX107/pDOP-αC; lane 4, pDOP-H3. Numbers at the bottom indicate the plasmid/chromosome ratio.

These constructs were introduced by mating into a *recA Rhizobium etli *CFN42 derivative lacking the p42d and p42a plasmids (CFNX107) (Figure 1). Only constructs pDOP-H3, pDOP-αC and pDOP-C were introduced with similar conjugation frequencies, from 1.6x10^-3 ^to 6x10^4^. However, CFNX107/pDOP-C transconjugants formed colonies after a longer time period (6-7 days), which was slower than the CFNX107/pDOP-αC and CFNX107/pDOP-H3 transconjugants and the receptor strain CFNX107 (3-4 days). Plasmid profile analyses of the transconjugants showed that the introduced plasmids replicated independently (Figure 2). The analyses also showed that pDOP-C replicated with a higher plasmid copy-number than pDOP-H3 carrying the complete p42d *repABC *operon. This observation was corroborated by measuring the plasmid copy-number of the transconjugants: 6 copies of pDOP-C were present per chromosome instead of 1-2 copies of the control plasmid pDOP-H3 (Figure 3).

**Figure 4:**
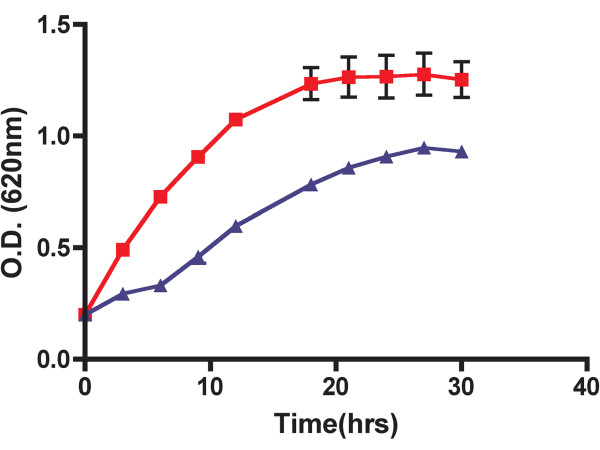
**Growth kinetics of *R. etli *CFNX107 (red line), and *R. etli *CFNX107/pDOP-C (blue line), in PY medium without antibiotics, incubated at 30°C, and 250 rpm (see Methods)**.

These results indicate that the minimal replicon of p42d consists of a *repC *gene under a constitutive promoter (Plac) and the SD sequence that we introduced and that the origin of replication resides within the *repC*-coding region. However, the growth rate of CFNX107 strain was negatively influenced by the introduction of pDOP-C (see Figure 4).

To prove that RepC is essential for replication, two *repC *deletions and two frame-shift mutants were constructed and cloned into pDOP under the control of the Plac promoter. Plasmid pDOP-C/D1UM contained a *repC *gene with a deletion of 14 codons (from codon 2 to 14), and plasmid pDOP-C/RD1L contained a *repC *gene with a deletion at its 3'end of 14 codons (from codon 388 to 401). The construct pDOP-CBglII possessed a *repC *gene with a frame-shift mutation at nucleotide 948, while plasmid pDOP-CSphI carried a frame-shift mutation at nucleotide 277. All of these constructs contained the same SD sequence as construct pDOP-C and were in the same relative orientation with respect to PLac in the vector. All plasmids were mated into the *R. etli *CFNX107 strain, but no transconjugants were obtained, indicating that the complete RepC product is crucial for replication.

To demonstrate that these observations were not specific to the p42d *repC *sequence, the *repC *genes of *S. meliloti *1021 pSymA and the *A. tumefaciens *C58 linear chromosome were amplified by PCR and introduced into pDOP under Plac control and downstream of a SD sequence. The recombinant plasmids were conjugated into *R. etli *strain CFNX107, and the plasmid profiles of the transconjugants were analyzed. Both recombinant plasmids were capable of replication in *Rhizobium*, as was pDOP-C (Figure 2). These results clearly suggest that the presence of an origin of replication (*oriV*) within *repC *is a general property of *repAB*C operons.

### Analysis of the *repC *sequence: the role of the high A+T content region

To circumscribe the origin of replication (*oriV*) of the *repABC *plasmids, we performed an *in silico *analysis to search for three sequence features that are characteristic of the *oriV *in low copy-number plasmids: a set of tandem direct repeat sequences (iterons), a region of high A+T content, and DnaA boxes. We only detected a region of high A+T content between positions 450 and 850 of the *repC *coding region. However, we did not find any trace of even highly degenerated direct repeat sequences or of DnaA boxes.

**Figure 5:**
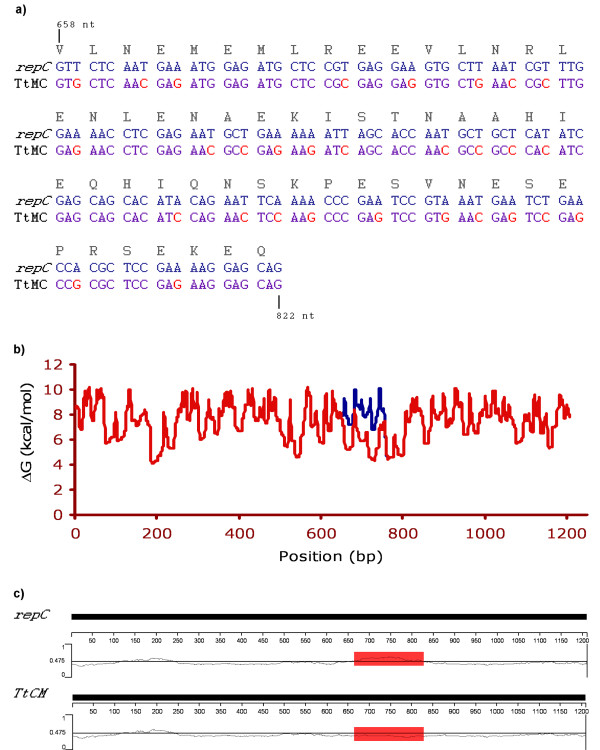
**a) Gene alignment of *repC *and and its mutant derivative pDOP-TtMC from position 658 to 822, indicating nucleotide changes introduced into pDOP- TtMC (red letters) to increase the C+G content of this region**. Note that the included mutations did not change the RepC protein sequence. **b) **DNA duplex stability expressed as ΔG along *repC *gene (red line) and its mutant derivative TtMC (blue line). **c) **Graphic showing A+T content along *repC *gene and its mutant derivative TtMC. A+T average in both genes is the same: 0.475. The A+T rich region of *repC *is boxed. Note that the equivalent region in TtMC, also boxed, the A+T content is above the average.

To determine if the high A+T content region has a role in plasmid replication, we constructed a *repC *derivative in which a group of silent mutations were introduced with the aim of altering the A+T content and increase the DNA duplex stability of this region, without disrupting the *repC *product (Figure 5). This *repC *mutant was cloned into pDOP under the Plac promoter and a SD sequence, generating the plasmid pDOP-TtMC. This plasmid could not replicate in *Rhizobium *strains with or without p42d, indicating that the A+T rich region plays a major role in replication.

### RepC exerts its action in *cis*

The identification of an *oriV *sequence is generally based on its ability to facilitate replication when present on a plasmid that otherwise could replicate only if the appropriate replication factors (e.g., an initiator protein) were provided in *trans*. To more precisely locate the *oriV *within *repC*, we cloned a collection of internal segments of *repC *into the suicide vector pDOP (Figure 1). This collection was conjugated into an *R. etli *strain containing the parental plasmid (CFNX101) as the source of all the *trans *elements required for replication, but we were unable to obtain transconjugants.

To determine if the activation of *oriV *requires transcription (i.e., the *repC *mRNA also acts as a replication primer), we constructed a pDOP derivative that contained a *repC *gene but lacked a SD sequence (pDOP-Cs/SD) (Figure 1). This plasmid was also incapable of replicating in *R. etli *CFNX101. Similarly, the two plasmids with *repC *frame-shift mutations, pDOP-CBglII and pDOP-CSphI, were also conjugated into *R. etli *CFNX101 without success. Overall, these results indicate that RepC exerts its action in *cis*.

### RepC as an incompatibility factor

Plasmid incompatibility, or the inability of two replicons to coexist in the same cell line, results from the sharing of elements involved in plasmid replication, partitioning or control [30]. The *repC *open reading frame of p42d, when cloned in a vector capable of replicating in *R. etli*, CFNX101, can coexist with p42d [8]. However, all of our attempts to introduce the construct pDOP-C into *R. etli *CFNX101 failed. In contrast, CFNX101 transconjugants carrying a similar construct (pDOP-CsA) that contained the *repC *gene pSymA of *S. meliloti *2011 were easily obtained. The frequencies with which CFNX101/pDOP-CsymA and CFNX107/pDOP-CsymA transconjugants were obtained were similar (average 5 × 10^-3^). Moreover, the plasmid profiles of the transconjugants showed that pDOP-CsA replicated in these strains as an independent entity. These observations indicate that pDOP-C and its parental plasmid p42d are incompatible, while that of pDOP-CSymA and p42d are compatible.

**Figure 6:**
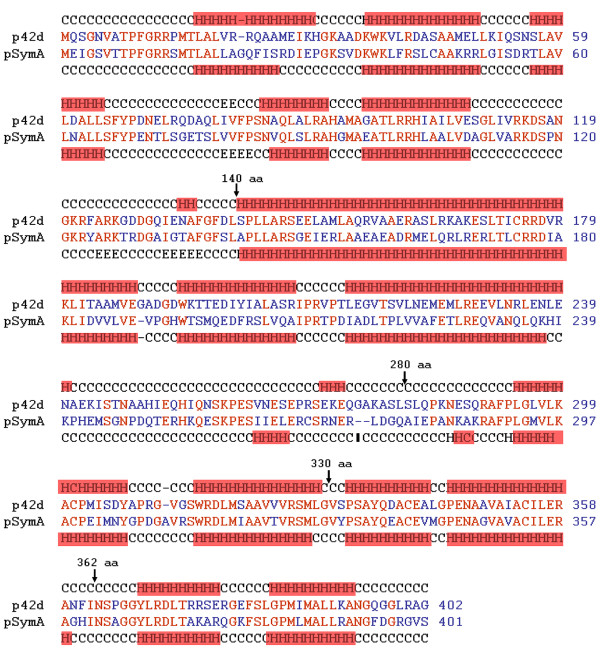
**Protein alignment of p42d RepC from *R. etli *CFN42 and pSymA RepC from *S. meliloti *1021 and where identical amino acid residues are marked in red**. The secondary structures of these proteins are also shown. Coiled regions are marked with C; helical regions are marked with boxed H letters; and with letter E, the stranded regions. Arrows with an associated numbers indicates the positions where the genes were swap, in the hybrid genes (see table 1).

**Figure 7:**
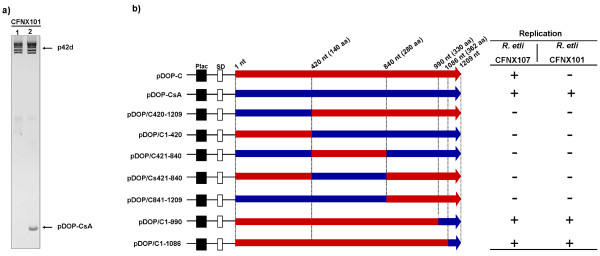
**a) Plasmid profiles of CFNX101 (lane 1) and CFNX101/pDOP-CsA (lane 2), showing that plasmid p42d and pDOP-CsA are compatible**. **b) **Linear representation of constructs containing SymA *repC *gene (blue arrow), p42d *repC *gene (red arrow) and SymA/p42d hybrid derivatives (blue/red arrows), and their associated replication capabilities when introduced into *R. etli *CFNX101 (with p42d) and CFNX107 (a p42d cured derivative) strains (table at left). "+" Symbols indicate that the construct are capable to replicate, and "-" that the construct is incapable to do that. Construct names are listed at the right of the figure. Black squares indicate the relative position of the Plac promoter, and the white rectangles the position of the Shine-Dalgarno (SD) sequences. Numbers at top indicate the positions where the SymA/p42d regions were swap.

The RepC protein of *S. meliloti *2011 pSymA shares 54% identity with the p42d RepC protein, and both proteins have very similar secondary structures (Figure 6). To map the RepC regions of p42d that are involved in plasmid incompatibility, a collection of hybrid genes containing fragments of the *repC *genes from *S. meliloti *pSymA and *R. etli *p42d were constructed. A schematic representation of the hybrid genes and their properties is shown in Figure 7. The hybrid genes were designed so that none of the predicted alpha-helix and beta regions of the *repC *products were disturbed. The hybrid genes were cloned into pDOP under the Plac promoter and transferred by conjugation into *R. etli *CFNX107 to determine their ability to replicate autonomously and into *R. etli *CFNX101 to test if they were able to replicate without the interference of p42d. Two constructs were capable of replicating in both genetic backgrounds: pDOP-C1-990 and pDOP-C1-1086. The rest of the constructs failed to replicate in both strains. The plasmid pDOP-C1-1086 expresses a hybrid protein containing the first 362 amino acid residues (aa) of the p42d RepC protein and the last 39 aa carboxy-terminal region of the pSymA RepC protein. With respect to plasmid incompatibility, this recombinant plasmid behaved the same as plasmid pDOP-CSymA, i.e., it replicated similarly in the strains CFNX101 and CFNX107. This result indicates that the RepC region involved in plasmid incompatibility resides in the last 39 amino acid residues of the protein.

## Discussion

Plasmids in which the *oriV *is located in the gene encoding an initiation protein are uncommon but not exceptional. The *Enterococcus faecalis *pheromone-responding plasmid pAD1 [31] (Francia, et al., 2004), the *Staphylococcus xylosus *plasmid pSX267 [32], the plasmids pAMβ1 and pLS32 from *Bacillus subtilis *[33-35], and the *Staphylococcus aureus *multiresistance plasmids pSK1 and pSK41 [36,37] fall into this category. However, the origins of replication in all of these plasmids have recognizable iterons, and an insert that contains some or all of the iterons from these plasmids is usually capable of driving plasmid replication if the initiator protein is provided *in trans*. The minimal replicon of the p42d plasmid is the *repC *ORF sequence driven by a constitutive promoter (Plac) with an SD sequence that we designed. Frame shift and deletion mutants of the *repC *gene disrupted the capacity for replication of the minimal replicon, indicating that RepC is essential for replication and is likely the initiator protein. To confirm this function, it will be necessary to demonstrate that this protein binds the *oriV*, melts the double-stranded DNA, and recruits the initiation host factor.

A DNA sequence analysis of the *repC *gene clearly showed the absence of iterons or other large, perfect or imperfect, repetitive sequences (>8 bp), which are the typical DNA-binding sites of plasmid initiator proteins [1].

The replication of several bacterial plasmids, such as P1, F, R6K, RK2, Rts1, pMU720, and pSC101, requires a crucial and concerted participation of DnaA and the plasmid-encoded initiator protein. These plasmids contain at least one DnaA box in their *oriV *sequences [38-43]. For other plasmids, DnaA participates only as an accessory, but these plasmids also contain DnaA boxes in their origins of replication (e.g., pR1) [44]. However, we failed to identify such DnaA boxes within the *repC*-coding region, suggesting that DnaA does not have a role in p42d replication.

A common property of theta-replicating plasmids is an A+T rich region close to the origin of replication, which is necessary for strand melting and the assembly of host initiation factors [1]. The *repC *ORF sequence of p42d contains a large A+T rich region that is crucial for plasmid replication. A construct carrying silent mutations that partially eliminated the A+T rich region was unable to promote replication in *R. etli *strains with or without the symbiotic plasmid, indicating that this region is an essential part of the *oriV*. However, a sequence analysis of other *repC *genes located in *repABC *operons revealed that an A+T rich region was present in all of the analyzed plasmids but its relative location was not conserved (data not shown).

The p42d minimal replicon (pDOP-C) has two intriguing properties. First, the construct resulted in enhancing the plasmid copy-number to around six, in contrast parental plasmid, which was maintained at 1-2 copies per chromosome. Second, the strain carrying this construct has a longer duplication time and a lower yield when the cells reach stationary phase than the strain without this construct.

While describing the observed increase in the plasmid copy-number, we must bear in mind that the *repC *gene in pDOP-C was expressed by a constitutive promoter.

In addition, the negative transcriptional regulation of the *repC *gene expression mediated by RepA and RepB was eliminated, and the antisense RNA (ctRNA), which also plays a negative role in the expression of *repC*, was removed. In the absence of these layers of negative regulation, it is expected that the plasmid replication would accelerate resulting in the production of new DNA molecules with a concomitant increase in the number of new origins of replication, which in turn, could be used to promote new rounds of replication, leading to cell death. However, in the present study, with the use of the minimal replicon (pDOP-C) we did not observe cell death, and the plasmid copy-number increased only moderately. This observation suggests the existence of a posttranslational mechanism that limits RepC activity, thus preventing over-initiation.

Growth kinetics of CFNX101 and CFNX107 were identical (data not shown), however, when pDOP-C was introduced into CFNX1017 growth of the bacterium was inhibited. The growth rate and yield diminution observed in strain CFNX107/pDOP-C relative to CFNX107 is not likely caused by the metabolic burden imposed by pDOP-C replication. The size of the parental plasmid (p42d) is approximately 374 Kb, while the size of pDOP-C is approximately 5.57 Kb; even if we take into consideration the 6-fold increase in plasmid copy-number, the amount of DNA required for replication in CFNX107/pDOP-C is several fold lower than the amount of DNA required for replication in CFNX101. Based on these observations it can be hypothesized that RepC, being an initiator protein, must perform three tasks: recognize the origin of replication, unwind the DNA at the origin, and recruit the replisome. An excess of RepC could lead to the formation of more of replication "bubbles". However, if one or more elements of the replisome are suboptimal in the growing cell, then, some replication forks will be stalled resulting in inhibition of cell division and growth.

We demonstrated that pDOP-C was capable of autonomous replication in an *R. etli *strain lacking the parental plasmid (p42d). However, we could not introduce this construct into an *R. etli *strain harboring the parental plasmid. In contrast, a similar construct that contained the *repC *gene of *S. meliloti *pSymA replicated autonomously with the same behavior in both strains. This result indicates that RepC is an incompatibility factor that prevents the coexistence of p42d and pDOP-C and that the incompatibility phenomenon is replicon-specific. Additionally, a construct (pDOP-C1-1086) expressing a chimeric protein consisting of the amino-terminal region of p42 RepC and 39 aa residues of the carboxy-terminal region of the pSymA RepC protein was capable of replicating as an independent entity with the same efficiency in *R. etli *strains, with or without p42d. This result indicates that the last 39 aa residues of the RepC carboxy-terminal region are directly involved in the incompatibility phenotype. A close inspection of this region in the RepC proteins of pSymA and p42d shows that they share 62.5% of identity, indicating that 15 amino acid residues or less are critical in promoting the incompatibility phenotype. Interestingly, however, in spite of the variations in 15 aa residues, RepC proteins of p42d and pSymA have a similar secondary structure: both possess two alpha helices of ten amino acid residues each, separated by a coiled region of six amino acid residues, in the same relative positions.

Our current hypothesis linking incompatibility and the RepC posttranslational regulation is as follows: RepC, like many other plasmid-encoded initiator proteins, exists in two forms, an active monomer and an inactive dimer, and protein thermodynamics favors dimer formation [1]. The RepC carboxy-terminal region is involved in dimer formation, and the dimerization process is replicon-specific. The introduction of pDOP-C into a strain containing p42d displaces the RepC monomer-dimer equilibrium that favors the inactive form, preventing the establishment of the incoming plasmid. A similar introduction of a construct with the RepC of a compatible plasmid will not affect the monomer-dimer equilibrium and will allow the establishment of the new plasmid.

Another unusual observation was the inability to complement the *repC *ORF *in trans *for replication. One possibility is that the *repC *transcript acts as an RNA primer for replication or assists in DNA melting at the *oriV*. However, the construct pDOP-Cs/SD, which lacks a SD sequence, could not replicate in CFNX101, suggesting that translation is required for the newly synthesized RepC protein to be located at the *oriV*. To the best of our knowledge, the only initiator protein that functions only in *cis *is RepA from prophage N15 [45]. At this stage we cannot determine which of these possibilities is more likely, and further experiments are needed to resolve these questions.

## Conclusions

RepC is the only element encoded in the *repABC *operon of the *Rhizobium etli *p42d plasmid that is necessary and sufficient for plasmid replication and is likely the initiator protein. The *oriV *of this plasmid resides within the *repC *gene and is located close to or inside of a large A+T region. This architecture is shared by other *repABC *plasmids. Our results also indicate that RepC can act as an incompatibility factor and that the last 39 aa of the carboxy-terminal region of this protein are involved in this phenotype.

## Competing interests

The authors declare that they have no competing interests.

## Authors' contributions

R C-R conducted the bulk of the experiments and made the constructions; F P-L and G P-S made growth kinetics, plasmid profiles and incompatibility experiments. MAC designed and coordinated the study, and wrote the manuscript. All authors read and approved the final manuscript.
